# Dinosaur Census Reveals Abundant *Tyrannosaurus* and Rare Ontogenetic Stages in the Upper Cretaceous Hell Creek Formation (Maastrichtian), Montana, USA

**DOI:** 10.1371/journal.pone.0016574

**Published:** 2011-02-09

**Authors:** John R. Horner, Mark B. Goodwin, Nathan Myhrvold

**Affiliations:** 1 Museum of the Rockies, Montana State University, Bozeman, Montana, United States of America; 2 Museum of Paleontology, University of California, Berkeley, California, United States of America; 3 Intellectual Ventures, Bellevue, Washington, United States of America; California Academy of Sciences, United States of America

## Abstract

**Background:**

A dinosaur census recorded during the Hell Creek Project (1999–2009) incorporates multiple lines of evidence from geography, taphohistory, stratigraphy, phylogeny and ontogeny to investigate the relative abundance of large dinosaurs preserved in the Upper Cretaceous Hell Creek Formation of northeastern Montana, USA. Overall, the dinosaur skeletal assemblages in the Hell Creek Formation (excluding lag-influenced records) consist primarily of subadult or small adult size individuals. Small juveniles and large adults are both extremely rare, whereas subadult individuals are relatively common. We propose that mature individuals of at least some dinosaur taxa either lived in a separate geographic locale analogous to younger individuals inhabiting an upland environment where sedimentation rates were relatively less, or these taxa experienced high mortality before reaching terminal size where late stage and often extreme cranial morphology is expressed.

**Methodology/Principal Findings:**

*Tyrannosaurus* skeletons are as abundant as *Edmontosaurus*, an herbivore, in the upper Hell Creek Formation and nearly twice as common in the lower third of the formation. Smaller, predatory dinosaurs (e.g., *Troodon* and dromaeosaurids) are primarily represented by teeth found in microvertebrate localities and their skeletons or identifiable lag specimens were conspicuously absent. This relative abundance suggests *Tyrannosaurus* was not a typical predator and likely benefited from much wider food choice opportunities than exclusively live prey and/or specific taxa. *Tyrannosaurus* adults may not have competed with *Tyrannosaurus* juveniles if the potential for selecting carrion increased with size during ontogeny.

**Conclusions/Significance:**

*Triceratops* is the most common dinosaur and isolated skulls contribute to a significant portion of this census. Associated specimens of *Triceratops* consisting of both cranial and postcranial elements remain relatively rare. This rarity may be explained by a historical collecting bias influenced by facies and taphonomic factors. The limited discovery of postcranial elements may also depend on how extensive a fossil quarry is expanded after a skull is collected.

## Introduction

The Hell Creek Project (1999–2009), a collaborative, multi-institutional field study of the Upper Cretaceous Hell Creek Formation, northeastern Montana, produced this dinosaur census from a well-documented collection of all taxa. The overall goal of the project was to create a comprehensive biotic foundation from which paleobiological and geological hypotheses could be tested. One of the many projects included the collection of dinosaur specimens to test hypotheses focusing on the relative abundances and the presence or absence of various dinosaurian ontogenetic stages. Previous Hell Creek Formation surveys [1], [2] attempted to statistically support particular extinction hypotheses, but offered minimal information on the actual composition of the stratigraphically dispersed assemblages through the entire section of the Hell Creek Formation. A dataset by Sheehan et al. [1] was used by White et al. [3] to examine the structure of the dinosaurian assemblage with regard to taphonomy only. Russell and Manabe [4] provided a clearer picture of relative abundances within the dinosaur assemblage, but failed to subdivide the Hell Creek Formation stratigraphically or to include ontogenetic perspectives in their analysis of previous surveys and collections. We think it is essential that all temporal and spatial points of reference be considered synthetically when analyzing taxa from the fossil record – a type of *unified field theory* for paleontological specimens. Unified frames of reference (UFR) include geography, taphohistory (defined here as the history of the specimen from death to final disposition within space and time), stratigraphy, phylogeny and ontogeny.

**Figure 1:**
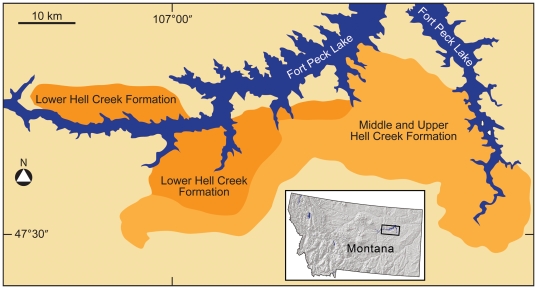
Index map of the Upper Cretaceous Hell Creek Formation along Ft. Peck Lake in northeastern Montana, USA. Contiguous outcrops are traceable over an area of about 1000 square km adjacent to Fort Peck Lake shown in blue. Dark orange represents the lower Hell Creek Formation and light orange represents the middle and upper Hell Creek Formation. The enlarged view of the study area is indicated by the rectangle in the northeast quarter of the map of Montana.

In this current survey, we focused on the Upper Cretaceous Hell Creek Formation exposed around Fort Peck Reservoir in northeastern Montana. Here, contiguous outcrops are traceable over an area of about 1000 sq. km (Figure 1). Facies changes were tracked within the formation's 90 to 100 meter thickness. Each confirmed skeleton was recorded and evaluated with regard to its UFR. This dataset is archived in the Museum of the Rockies (MOR).

Here we present the relative abundance of large dinosaur taxa, their general ontogenetic stage within a stratigraphic and sedimentological context and offer generalized hypotheses to explain several of the more interesting patterns that have emerged. This dinosaur census sets the stage for future statistical analysis and evaluation of the end Cretaceous faunal record and will add to recent paleoecological studies of diverse dinosaur faunas and clades that focused on body size, habitat partitioning and living space requirements [5] or abundance modeling [6].

The Hell Creek Project spanned 11 field seasons from 1999–2009. The first five years focused on the collection of specimens from the lower third of the formation (See Figure 1, dark orange area), while the remaining six years were aimed at specimen collection from the middle and upper strata (Figure 1, light orange area). Since several of the dinosaur specimens from the most recent field seasons (2008–2010) remain unprepared, the data presented here for the upper two-thirds of the formation will be updated as Hell Creek Project specimens are prepared and available from the corresponding author and on www.museumoftherockies.org.

## Methods

### Geological Methods

**Figure 2:**
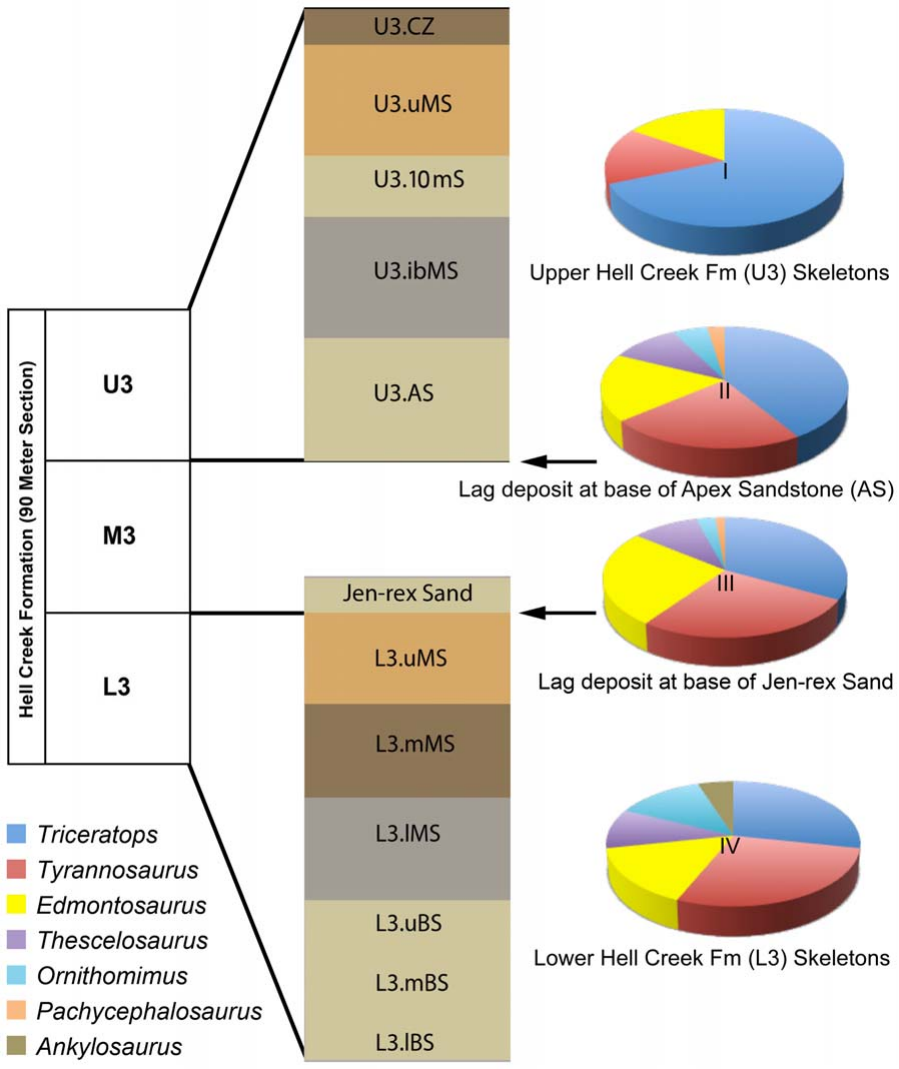
Stratigraphic divisions of the Hell Creek Formation with stratigraphic details of the upper third (U3) and lower third (L3) sequences referenced in the text and the associated pie chart showing dinosaur abundance. Pie charts I – IV reflect the relative abundance of dinosaur genera based on skeletons (charts I and IV) and individual bones (charts II and III). *Tyrannosaurus* skeletons are as abundant as the hadrosaurid *Edmontosaurus*, an herbivore, in the upper Hell Creek Formation and nearly twice as common as *Edmontosaurus* in the lower Hell Creek Formation. Individual bones of *Tyrannosaurus* and *Edmontosaurus* are found approximately in the same order of magnitude within the lag deposits. **Key:** **Pie chart I**, dinosaur skeletons in the upper Hell Creek Formation (U3); **Pie chart II**, dinosaur bones from the “Doldrum’s” lag deposit at the base of the Apex Sandstone (AS); **Pie chart III**, dinosaur bones from the “3B-1” lag deposit at the base of the Jen-rex Sand; and **Pie chart IV**, dinosaur skeletons from the lower Hell Creek Formation (L3). **Abbreviations:** **L3lBS**, lower basal sandstone; **L3.mBS**, middle basal sandstone; **L3.uBS**, upper basal sandstone; **L3.lMS**, lower mudstone; **L3.mMS**, middle mudstone; **L3.uMS**, upper mudstone; **U3.AS**, Apex sandstone; **U3.ibMS**, interbedded mudstone; **U3.10mS**, 10 meter sandstone; **U3.uMS**, upper mudstone; **U3.CZ**, coal zone.

In order to evaluate possible changes in dinosaur taxa and their relative abundance through time, the Hell Creek Formation was divided into three stratigraphic units [7], [8] designated L3 (lowest), M3 (middle) and U3 (upper) (see Figure 2). Only specimens from the L3 and U3 are included in this census because these units show the greatest faunal contrast and have the highest resolution from sedimentological and stratigraphic controls in contrast to M3. A continuing evaluation of the geology and paleontology of M3 will be reported on in a follow-up study by the senior author and the Hell Creek Project team.

In this study, the L3 and U3 are subdivided based on sedimentological and stratigraphic details confirmed in the study area (Figure 1). The L3 basal sandstone (L3.BS) is further subdivided into three stratigraphic horizons: lower, L3.lBS; middle, L3.mBS; and upper, L3.uBS. An overlying variegated mudstone (L3.MS) is also subdivided into three traceable units: lower, L3.lMS; middle, L3.mMS; and upper, L3.uMS. Because a dinosaur – bearing lag deposit was not identified in the lower third (L3) of the Hell Creek Formation, we use the Jen-rex sand [7] at the base of M3 as a proxy for this facies in L3 because of its stratigraphic position along the M3–L3 contact (see Figure 2).

The U3 is comprised of two sandstone units: the Apex sandstone (U3.AS) and the 10-meter sandstone (U3.10mS). An interbedded variegated mudstone (U3.ibMS) separates these two sandstones and an upper variegated mudstone (U3.uMS) overlies the 10-meter sandstone. U3 is capped by an overlying uppermost coal zone (U3.CZ).

High-resolution digital video (remote control camera mounted on a Bell 407 helicopter) was utilized to track stratigraphic horizons and facies over large distances to assure stratigraphic resolution and supplement standard stratigraphic sections and mapping within the study area.

### Census Methods

Census figures for the dinosaurs were determined using two different methods: (1) recording numbers of articulated or associated skeletons and (2) recording numbers of individuals based on isolated elements collected from lag deposits in the L3 ( = Jen-rex sand) and U3 ( = Apex sandstone). Although more than 150 microsites were recorded and sampled during the course of the Hell Creek Project, microsite census data are not included here because we think these sites impart biased dinosaur census data (contra [2]) because of the limited size of microsite specimens due to facies and size sorting dynamics of the particular hydrologic regime. While the shed teeth of adult dinosaurs such as *Triceratops, Edmontosaurus, Thescelosaurus, Pachycephalosaurus*, and *Ankylosaurus* are commonly found in microsites, the adult shed teeth of *Tyrannosaurus* are very rare, likely because they are much larger and heavier than the specimens commonly recovered from these deposits. In addition, the microsites sampled by the MOR crews were surface collected rather than screened, and could therefore potentially impart a collector-biased faunal record (e.g., we observed that small theropod teeth are often surface collected in greater numbers due to either a taphonomic or collector bias, or both, compared to ornithischian teeth). Nonetheless, these stratigraphically constrained microsites provide a basis for future comparisons with similar Mesozoic ecosystems [9]–[11].

Countable dinosaur specimens are herein defined as any group of three or more bones most likely belonging to one individual based on size, association and taphonomic details (color, wear, etc.). We assume that three bones represent the minimum number of elements of a skeleton or skull that has not been transported and redeposited after initial burial. As for bones collected from lag deposits, it is assumed that these sites contain a potentially time-averaged, cross section of the dinosaur fauna, as long as the skeletal elements vary in size from small teeth to large limb elements (≥0.50 m). Census results from two such deposits are included in this study: (1) the Jen-rex sand (3B-1 Lag: MOR locality no. HC-312) and (2) the U3 unit at the base of the Apex sandstone (Doldrum's Lag: MOR locality no. HC-530). Lag specimens of dinosaurs were collected and logged only if they could be reasonably identified to genus. Dinosaur genera recognized for this study include *Triceratops* (*Torosaurus* and *Nedoceratops* have been synonymized with *Triceratops* [12]), *Edmontosaurus* (“*Anatotitan*” is not recognized in this area), *Thescelosaurus* (*Bugenosaura* has been synonymized with *Thescelosaurus* [13]), *Pachycephalosaurus* (*Stygimoloch* and *Dracorex* have been synonymized with *Pachycephalosaurus* [14]), *Ankylosaurus, Ornithomimus, and Tyrannosaurus* (*Nanotyrannus* is considered a juvenile *Tyrannosaurus* [15]). No other dinosaurian material represented taxa we could identify to genus.

In addition to the census of skeletons and lag specimens (Tables S1,S3, S4, S5, S6) we also provide a list of *Triceratops* skulls (Table S2) that were located in the L3 unit, recorded, but not collected. Here, specimen collection varied according to the year of discovery. *Triceratops* specimens located in the early years (1999–2003) of the Hell Creek Project were not collected unless it was obvious that associated, disarticulated elements were present. If we could not determine the degree of articulation to indicate a skeleton by simple surface observation, the specimens were recorded and left in-situ. We later learned that extensive excavation was required to make this evaluation. This “learning process” probably explains the majority of the differences in *Triceratops* abundance between the census numbers from the L3 (n = 11) and the U3 (n = 22) units. When weathered *Triceratops* skulls were located during the first five-year period, their positions were recorded without excavation, even though it was clear that each of the skulls consisted of at least three distinct cranial elements (e.g., postorbital horn, squamosal and jugal). During the latter years of the project following 2003, every *Triceratops* specimen found was excavated regardless of how eroded or poorly preserved the exposed bones first appeared to be. Upon excavation, most specimens were found disarticulated with fair to very good preservation at depth. Those occurrences representing a countable skeleton were logged into the skeletal census while specimens that did not meet these criteria were not.

With the exception of one specimen of *Tyrannosaurus rex* (designated “N-rex”) curated into the Smithsonian Institution's National Museum of Natural History, all the dinosaur specimens in this study are curated into the paleontology collections of the Museum of the Rockies, Montana State University, Bozeman, MT, USA.

### Ontogenetic Methods

In addition to producing a census of individuals of each taxon in the L3 and U3 units, we also determined the relative ontogenetic stages of the counted individuals. These general ontogenetic stages were determined by morphological ontogenetic markers in some taxa, such as *Triceratops* [16], using ontogenetic histological methods for others, such as *Tyrannosaurus* [17], or using a combination of these techniques for taxa such as *Pachycephalosaurus* [14].

Six ontogenetic stages identified with a letter ascending in size from “A” to “F” are employed for the *Triceratops* skeletal material (Tables S1). *Triceratops* has the widest published ontogenetic range [12], [16] and therefore allows for higher ontogenetic resolution than other taxa. Each letter corresponds to a relative age class: “A,” represents small juveniles; “B,” large juveniles; “C,” small subadults; “D,” large subadults; “E,” small adults; and “F,” large adults. These stages are simply determined relative to one another based on the smallest and largest end member skulls. Within specimens of *Triceratops*, for example, “A” individuals have skulls approximately 0.3 m in length; F-size skulls are approximately 3.0 m in length. Letters in-between “A” and “F” provide a general sense of intervening sizes. These stages are useful for the purpose of demonstrating the rarity or abundance of certain size classes of individuals and communicating these occurrences. Four size ranges are utilized for taxa other than *Triceratops* and for all the isolated fossils from the lag deposits (Tables S3, S4, S6): small “S”, medium “M,” large “L,” and extra-large “XL”.

## Results

### Geological Results

*Edmontosaurus*, *Ornithomimus* and *Ankylosaurus* are found in siltstones or sandstones, and *Thescelosaurus* is found exclusively in mudstones, but the relative number of specimens is small and subsequently questionable as a real pattern of sediment preference or taphonomic artifact (Tables S1, S4). Other taxa are found in both channel and overbank sediments, but the majority of *Triceratops*, and in particular juvenile specimens, come primarily from mudstones [18]. There was no apparent sediment preference for preserving articulation. The basal sand unit (L3.lBS) produced both an articulated specimen of *Edmontosaurus* (“X-rex”/MOR 1142) and a disarticulated *Tyrannosaurus* (“B-rex”/MOR 1125). An articulated *Tyrannosaurus* (“N-rex”/Smithsonian Institution) and a disarticulated *Tyrannosaurus* (“G-rex”/MOR 1128) were found in the lower mudstone unit (L3.lMS).

### Census Results

The dinosaur census results are summarized in Table 1 by taxon with percentage of the fauna and absolute numbers given. Additional sedimentological details, more precise stratigraphic interval, preservation and ontogenetic designations are provided in Tables S1, S2, S3, S4, S5, S6. The isolated, uncollected *Triceratops* skulls listed in Table S3 are not included in the census of skeletons from the lower Hell Creek Formation (Table S1) at present because there is no way to know if they consist of three or more disarticulated pieces until they are collected. Thirty-nine skeletons (not counting the isolated *Triceratops* skulls) were recorded from the L3 strata. All but three of these skeletons were collected. The uncollected specimens were represented by at least three elements but were too severely eroded to yield data other than for this census. In addition, seven specimens (superscript^3^ numbers in Table S1) consisted of only three elements each. Limited excavation around the elements failed to yield more material, and the sites were abandoned. Five specimens were found with some articulation, and of these, only one (*Edmontosaurus*, “X-rex,” MOR 1142) was found with skin impressions.

**Table 1:** Hell Creek Formation dinosaur census.

					Taxon				
Stratigraphic level		*Tric*	*Tyrn*	*Edmn*		*Thes*	*Orni*	*Pachy*	*Anky*
Upper Hell Creek Fm (U3) skeletons	n = %	2369%	516%	516%					
Pie chart I, Fig. 2 Table S4									
"Doldrum’s" lag deposit at base of Apex sandstone (MOR locality HC-530)	n = %	1641%	923%	718%		410%	25%	13%	
Pie chart II, Fig. 2 Table S6									
"3B-1" lag deposit at base of Jen-rex sand (MOR locality HC-312)	n = %	2333%	1927%	1826%		710%	23%	11%	
Pie chart III, Fig. 2 Table S3									
Lower Hell Creek Fm (L3) skeletons	n = %	1128%	1128%	615%		410%	513%		25%
Pie chart IV, Fig. 2 Table S1									
Totals for the entire Hell Creek Formation(see Figure 4)	n = %	7340%	4424%	3620%		158%	95%	21%	21%

Values determined from the dinosaur census tables (Tables S1, S2, S3, S4, S5, S6). Empty cell indicates no record for that taxon. See Figure 2 for detailed stratigraphic section of the Hell Creek Formation and corresponding pie chart showing relative abundance of dinosaur genera. Abbreviations: *Tric, Triceratops; Tyrn, Tyrannosaurus; Edmn, Edmontosaurus; Thes, Thescelosaurus; Orni, Ornithomimus; Pachy, Pachycephalosaurus; Anky, Ankylosaurus.*

The most interesting census result in the L3 is the high number of *Tyrannosaurus* skeletons (n = 11) that is nearly double the number of *Edmontosaurus* skeletons (n = 6) and equals *Triceratops* (n = 11) (see Figure 1 and Table 1). However, as explained in the previous paragraph, it is likely the number of *Triceratops* skeletons will increase as these sites in L3 are excavated. *Tyrannosaurus* contributes to 28% of the dinosaur skeletons recorded in L3 while *Edmontosaurus* makes up only 15%. Considering the fact that *Tyrannosaurus*, *Triceratops*, and *Edmontosaurus* are all relatively similar in size as full-grown adults, we presume that there are few taphonomic biases that would amplify the *Tyrannosaurus* numbers to be greater than *Edmontosaurus* and this likely reflects a correct ratio of approximately 2∶1.

Thirty-two skeletons were collected from the U3 unit, four of which were collected prior to the Hell Creek Project by the Museum of the Rockies (MOR 009, *Tyrannosaurus*; MOR 004, *Triceratops*; MOR 555, *Tyrannosaurus*; MOR 622, *Triceratops*; MOR 007, *Edmontosaurus*). These were included in the census because they were found in the study area with documented stratigraphic and locality information, and they are cataloged into MOR. *Triceratops* skeletons (n = 22) greatly outnumbered other taxa (Figure 2, Table 1) and contribute to 69% of the total dinosaur skeletal fauna in U3. Specimens of *Ornithomimus, Thescelosaurus*, *Ankylosaurus* or *Pachycephalosaurus* were conspicuously absent, although isolated bones of *Thescelosaurus, Ornithomimus*, and *Pachycephalosaurus* were present in the Doldrum's Lag deposit (MOR loc. HC-530) at the base of the Apex sandstone. *Edmontosaurus* and *Tyrannosaurus* skeletons were equal in number (n = 5) in U3 and comprise 16% each of the large dinosaur taxa. The pie charts in Figure 2 illustrate the similarity in overall percent composition between the large dinosaur fauna recorded in L3 and the two overlying lag deposits. The greatest contrast occurs within the upper Hell Creek (U3) record of dinosaur skeletons where *Triceratops* dominates (69%; n = 22), followed by *Tyrannosaurus* (16%; n = 5) and *Edmontosaurus* (16%; n = 5).

**Figure 3:**
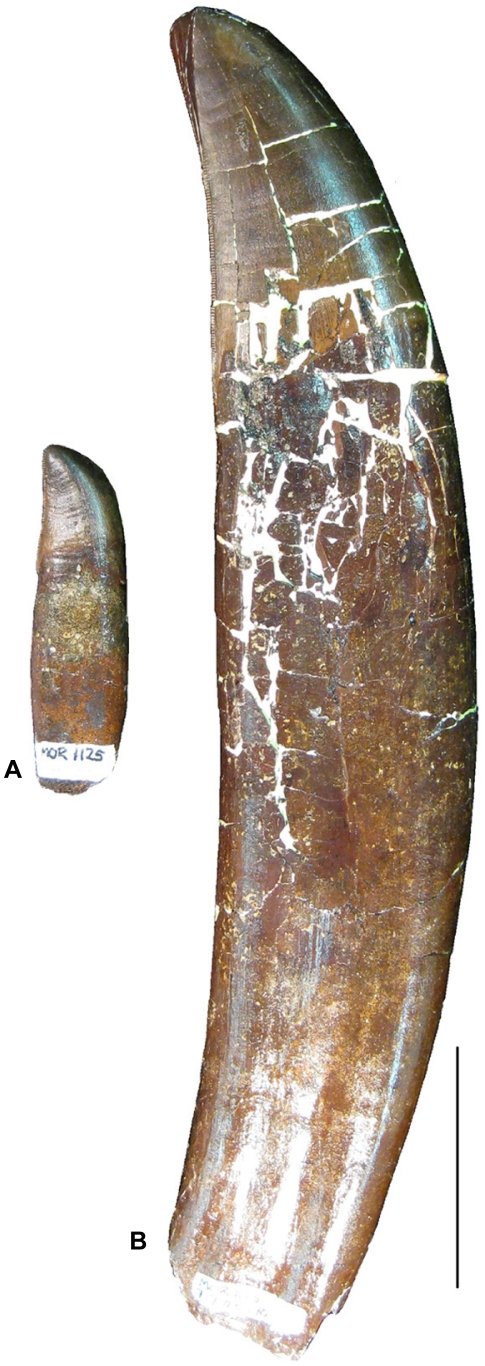
*Tyrannosaurus* (MOR 1125, “B-rex”) teeth from the lower jaw of this medium-sized skeleton illustrate the extreme range in overall tooth size within one individual. **A.** A smaller posterior tooth from position #14 from the front of the jaw. **B.** A larger tooth from position #4 in the same jaw. This demonstrates why shed dinosaur teeth are not a reliable indicator of relative skeletal size and ontogenetic age.

Teeth were not collected or annotated because of the difficulties in using them for ontogenetic assessment with the exception of two large *Tyrannosaurus* teeth from the “3B-1 Lag” at the base of the Jen-rex sand. Tooth size varies as much as 300% in a single jaw, particularly in hadrosaurids (MOR 1609, Becky's Giant), ceratopsids (MOR 2574, Quittin Time) and tyrannosaurids (MOR 1125, B-rex). This is one reason the assignment of some dinosaur teeth to “babies” [19] may be incorrect. These teeth are more accurately interpreted as being derived from the anterior or posterior portions of jaws from older individuals (Figure 3). Only the largest and most robust tyrannosaurid teeth are reliable indicators of adults.

The *Triceratops* specimens recorded in Table S6 represent specimens that were collected, but remain unprepared, uncataloged and consist of an unknown number of disarticulated elements.

### Ontogenetic Results

In this census, growth stages at either end of the dinosaurian ontogenetic spectrum are least represented. Specimens of both the smallest and presumably youngest juveniles and the largest, and presumably oldest adults are the most rare dinosaurs recorded. The smallest specimen of *Triceratops* found during this project is a partially complete skull that is half again the length of the smallest previously known skull [20]. None of the *Triceratops* specimens found in the census area could be positively identified as “*Torosaurus*” size, although the specimen collected from the “BAB” locality has elongated squamosals characteristic of the “*Torosaurus*” morph. Two specimens of *Edmontosaurus* are in the “XL” size range: “Becky's Giant” (MOR 1609) is a maxilla with a tooth-row length of 570 mm and the tail of “X-rex” (MOR 1142) is 7.5 meters in length from the posterior end of the sacrum. Both these specimens are indicative of greater size ranges then previously attributed to *Edmontosaurus*.

## Discussion

### Census

**Figure 4:**
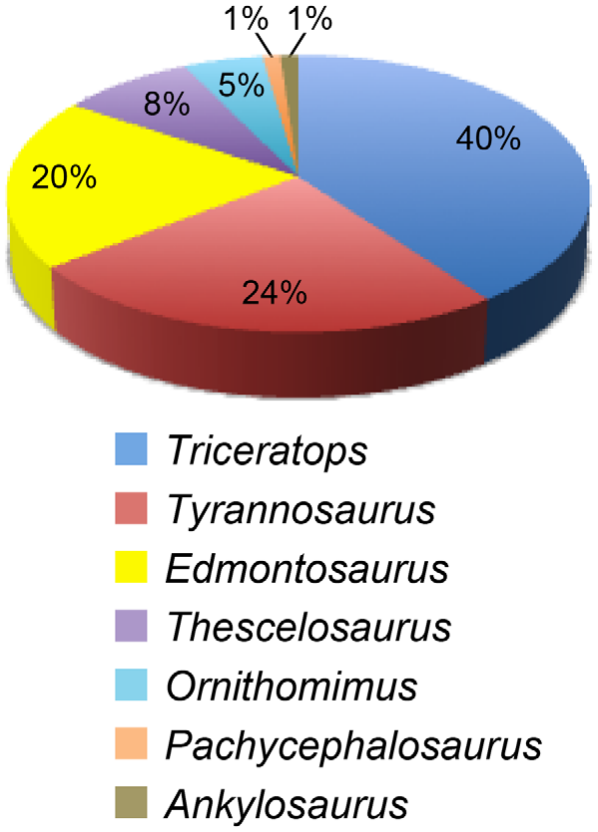
Pie chart of the time averaged census for large-bodied dinosaurs from the entire Hell Creek Formation in the study area. *Triceratops* is the most common dinosaur at 40% (n = 72); *Tyrannosaurus* is second at 24% (n = 44); *Edmontosaurus* is third at 20% (n = 36) followed by *Thescelosaurus* at 8% (n = 15), *Ornithomimus* at 5% (n = 9), and *Pachycephalosaurus* and *Ankylosaurus* at 1% (n = 2).

The dinosaur collections made over the past decade during the Hell Creek Project yielded new information from an improved genus-level collecting schema and robust data set that revealed relative dinosaur abundances that were unexpected, and ontogenetic age classes previously considered rare. We recognize a much higher percentage of *Tyrannosaurus* (Table 1) than previous surveys [3], [4], [21]. *Tyrannosaurus* equals *Edmontosaurus* in U3 and in L3 comprises a greater percentage of the large dinosaur fauna as the second most abundant taxon after *Triceratops*, followed by *Edmontosaurus*. This is surprisingly consistent in (1) the two major lag deposits (MOR loc. HC-530 and HC-312) in the Apex sandstone and Jen-rex sand (Figure 2) where individual bones were counted and (2) in two-thirds of the formation reflected in L3 and U3 records of dinosaur skeletons only. Measured throughout the entire formation, *Triceratops* is by far the most common dinosaur at 40% (n = 72), *Tyrannosaurus* is second at 24% (n = 44), *Edmontosaurus* is third at 20% (n = 36), followed by *Thescelosaurus* at 8% (n = 15), *Ornithomimus* at 5% (n = 9), and *Pachycephalosaurus* and *Ankylosaurus* both at 1% (n = 2) are relatively rare (see Figure 4).

Even though *Triceratops* dominates this census, associated specimens of *Triceratops* consisting of both cranial and postcranial elements remain relatively rare (see Tables S1, S2). This contrasts with the record of isolated skulls that contribute to a significant portion of this census. We propose that this inconsistency may be explained by a historical collecting bias influenced by taphonomic controls. This is documented in museum collections [18]. Alternatively, predation, scavenging, or some as yet unknown vital effect of rapid deterioration of *Triceratops* limb elements may limit their preservation in the fossil record. We observed that postcranial elements are often located at some distance from the associated skull, particularly in the preservation of *Triceratops*. Thus, the limited discovery of postcranial elements may, in some circumstances, simply depend on how extensive a quarry is expanded after a skull is collected.

### Ontogenetic Stages

When ontogenetic stages are considered, we observe a low number of both “A” and “F” class (see ontogeny column in Tables S1, S3, S4, S6) of *Triceratops* individuals and “S” and “XL” individuals of other taxa. Overall, the dinosaur assemblages represented in the Hell Creek Formation consist primarily of subadult or small adult size individuals (based on comparisons with the largest specimens of known taxa). Small juveniles and large adults are both extremely rare, whereas subadult individuals (M & L and D & E) are relatively common. The paucity of juveniles seen in the Hell Creek Formation and contemporaneous sediments puzzled earlier researchers [22]. This can likely be explained by a combination of: (1) extended parental care [23]–[25]; (2) rapid juvenile growth [26], [27]; and (3) colonial nesting in select geographic environments [19], [28]. This pattern likely reflects either a preservational (taphonomic) or life history consequence acting on the dinosaur population.

The uncommonness of apparently fully mature adults is more mysterious and not easily explained. What is now apparent, however, is this pattern contributed to an historical increase in the naming of new dinosaur species from the Hell Creek Formation. For example, over many decades it was presumed that the taxon “*Torosaurus*” represented a horned dinosaur that reached enormous proportions, even though there were no reported juveniles in the literature. The relatively expanded and fenestrated parietosquamosal frill exhibited by “*Torosaurus*” was among its most significant features [29]. With the advent of studies employing ontogenetic osteohistology, the alternative hypothesis that these giant dinosaurs were more likely mature individuals of existing taxa, rather than distinct taxa, became evident. This hypothesis is exemplified in recent studies of *Triceratops* ontogeny [12], [30] that reinterpret “*Torosaurus*” as an adult *Triceratops*. Nonetheless, this hypothesis fails to explain why these giant, mature individuals are so rare, or more explicitly, why most *Triceratops* specimens are subadult sized. We propose that mature individuals of at least some dinosaur taxa either lived in a separate geographic locale analogous to younger individuals inhabiting an upland fauna, or these taxa experienced high mortality rates before reaching terminal size where late stage and often extreme cranial morphology is expressed.

Reproductive maturity in some dinosaurs was achieved during subadulthood (e.g., *Tyrannosaurus*, *Allosaurus* and *Tenontosaurus*) and this event led to high adult mortality [31]. Interestingly however, our census data indicate the highest mortality occurred when *Triceratops* was about 2/3 grown ( = skulls approximately 2.0 m in length compared to adults with 3.0 m long skulls) prior to the final ontogenetic stage of frill expansion and fenestration in *Triceratops* ( = “*Torosaurus*”). *Edmontosaurus* conforms to a similar scenario where the “XL” size individuals are the most rare, and the mid-size (“M” and “L”) individuals are the most common. This pattern is difficult to evaluate in *Tyrannosaurus* because of apparent variations in age relative to size [17]. Nonetheless, we predict a larger specimen of *Tyrannosaurus* than currently known will likely be discovered in future field studies. Although the lines of arrested growth (LAGs) observed in the largest yet known *Tyrannosaurus* specimens [17] suggest slowed growth, and therefore a presumed nearing of maturity, the cortex tissues of the femora and tibia of these individuals remain mostly primary. This contrasts with the femoral and tibial cortex tissues of the largest individuals of *Triceratops* and *Edmontosaurus* that are mostly secondary (dense Haversian), which is a much more mature form of cortical tissue. This suggests that *Tyrannosaurus* growth would have continued, resulting in a bulking-up of the skeleton by continued additions of periosteal bone tissues, possibly to the external fundamental system (EFS), which signifies maturity in other taxa [26].

### *Tyrannosaurus* Abundance

The abundance of *Tyrannosaurus* specimens both as skeletons and as isolated elements in the LAG deposits contradicts hypotheses concerning predator-prey ratios expected for large, predatory terrestrial animals such as tyrannosaurids [32], [33]. Although constant ratios are suspect in modern ecosystems [34], [35], there are always at least 75% more non-predators than predators, and in mammal populations the ratio is >90% [32 and references therein]. What is particularly interesting in this census is the indication that *Tyrannosaurus* is at least as abundant in the upper Hell Creek Formation as *Edmontosaurus*, an herbivore, previously suggested to be the primary food source of *Tyrannosaurus* [36] (Figure 2). In the remaining two-thirds of the formation, *Tyrannosaurus* is more plentiful than *Edmontosaurus* (Table 1). Because the smaller, predatory dinosaur taxa *Troödon* and dromaeosaurids (known from teeth found in microsites) are extremely rare (no skeletons or identifiable lag specimens), it stands to reason that *Tyrannosaurus* was not a typical predator [37]. In fact, the large numbers of *Tyrannosaurus* compared to the smaller theropods suggest that *Tyrannosaurus* benefited from much wider food choice opportunities than exclusively live prey and specific taxa such as *Edmontosaurus* [36]. A similar comparison can be made with mammal census numbers from the Serengeti plains where the hyena population is twice that of the combined population of lion, leopard and cheetah [38], [39]. *Tyrannosaurus* may have acquired a larger percentage of meat from carrion sources than did smaller theropods, therefore filling the role of a more generalized, carnivorous opportunist such as a hyena. Based on energetic arguments [40], a Serengeti type ecosystem would have provided ample carrion to feed a *Tyrannosaurus* sized scavenger, particularly if *Tyrannosaurus* did not have to compete with avian scavengers. In addition, *Tyrannosaurus* adults may not have competed with *Tyrannosaurus* juveniles if the potential proclivity for carrion increased with size during ontogeny [41], [42]. Such a situation might well explain why *Tyrannosaurus* teeth increase in overall robustness while the total number of teeth in the lower jaws decrease during late stages of ontogeny [15].

### Conclusions

The Hell Creek Project generated eleven years (1999–2009) of collecting and field studies in the Upper Cretaceous Hell Creek Formation, eastern Montana, and resulted in the discovery of a wide variety of new dinosaur specimens, many of which revealed for the first time the ontogeny of well-known dinosaur taxa such as *Triceratops*. More than 240 associated or articulated dinosaur specimens were collected or recorded, and of these, smaller juveniles or larger adults were underrepresented, suggesting that these size individuals were not as common in the Hell Creek ecosystem. Nesting horizons in broadly contemporaneous formations suggest juveniles probably lived in other locations and the largest adults may have simply been relatively rare within their populations. Mortality rates appear to be higher among individuals that were not yet fully mature. This may have resulted in positive feedback acting on earlier maturation rates during ontogeny in some dinosaurs.

The relatively high abundance of *Tyrannosaurus* contradicts earlier suggestions that it was a very rare taxon in the Hell Creek Formation [21]. This census suggests that *Tyrannosaurus* was not strictly a predator, but instead more of an opportunistic feeder, possibly selecting similar food choices under circumstances comparable to that of hyenas in extant ecosystems, a trend unrecognized in earlier census studies.

## Supporting Information

Table S1 **Lower Hell Creek Formation (L3) dinosaur skeletons in order of abundance.**
(DOC)

Table S2 **Upper Hell Creek Formation (U3)** ***Triceratops*** **skulls collected.** These specimens are either whole skulls or isolated elements and do not qualify to be counted under the “three bone rule.”
(DOC)

Table S3 **Dinosaur census from 3B1 lag deposit (MOR loc. HC-312) at the base of the Jen-rex sand in order of abundance.**
(DOC)

Table S4 **Upper Hell Creek Formation (U3) skeletons in order of abundance.**
(DOC)

Table S5 **Upper Hell Creek Formation (U3)** ***Triceratops*** **skulls recorded but not collected.**
(DOC)

Table S6 **Census from the Doldrum's lag deposit (MOR loc. HC-530) at the base of the Apex sandstone in the upper Hell Creek Formation (U3.AS) in order of abundance.**
(DOC)
