# Supplementary material for: Dinosaur Census Reveals Abundant Tyrannosaurus and Rare Ontogenetic Stages in the Upper Cretaceous Hell Creek Formation (Maastrichtian), Montana, USA
Source: PLoS One. 2011 Feb 9;6(2):e16574. doi: 10.1371/journal.pone.0016574 (PMC3036655; doi:10.1371/journal.pone.0016574)
Supplement: Table S1 — Lower Hell Creek Formation (L3) dinosaur skeletons in order of abundance. (DOC) [file pone.0016574.s001.doc]

| **Taxon** | **Loc. Name** | **Loc. No.** | **MOR No.** | **Sed.** | **Strat.** | **Pres.** | **Onto.** |
| --- | --- | --- | --- | --- | --- | --- | --- |
| *Triceratops* | Getaway Trike | HC-256 | MOR-1120 | ms | L3.mMS | assoc | D |
| *Triceratops* | Independence | recorded | uncollected | ms | L3.mMS | assoc | D |
| *Triceratops* | Mark's Trike | HC-308 | MOR-1186 | ms | L3.mMS | assoc | E |
| *Triceratops* | BOSH | HC-393 | MOR-2552 | ms | L3.mMS | assoc | E |
| *Triceratops* | Otta Water | recorded | uncollected | ms | L3.lMS | assoc | E |
| *Triceratops^3^* | Celestial | HC-249 | MOR-1109 | ms | L3.lMS | assoc | E |
| *Triceratops* | BH-003 | HC-283 | MOR-1157 | ms | L3.lMS | assoc | E |
| *Triceratops* | Heaven | HC-285 | MOR-1160 | ms | L3.lMS | assoc | E |
| *Triceratops* | Triceratopolis | HC-310 | MOR-1188 | ms | L3.lMS | assoc | E |
| *Triceratops* | 2 Big | HC-575 | MOR-2984 | ms | L3.lMS | assoc | E |
| *Triceratops* | Bill's Trike | HC-X01.2 | MOR.P01.1 | sls | L3.uBS | assoc | E |
| *Tyrannosaurus^3^* | Jen-rex | HC-316 | MOR-1198 | ss | L3.BL | assoc | L |
| *Tyrannosaurus* | L-rex | HC-263 | MOR-1127 | ms | L3.uMS | assoc | M |
| *Tyrannosaurus* | F&H Theropod | HC-447 | MOR-2600 | ms | L3.mMS | assoc | M |
| *Tyrannosaurus* | C-rex | HC-262 | MOR-1126 | ms | L3.mMS | **artic** | XL |
| *Tyrannosaurus* | J-rex-1 | HC-266 | MOR-1151 | ms | L3.lMS | assoc | M |
| *Tyrannosaurus* | N-rex | HC-285 | NMNH | ms | L3.lMS | **artic** | M |
| *Tyrannosaurus* | G-rex | HC-264 | MOR-1128 | ms | L3.lMS | assoc | L |
| *Tyrannosaurus^3^* | J-rex-2 | HC-282 | MOR-1156 | Ms | L3.lMS | assoc | L |
| *Tyrannosaurus* | F-rex | HC-280 | MOR-1152 | ms | L3.uBS | assoc | XL |
| *Tyrannosaurus* | B-rex | HC-261 | MOR-1125 | ss | L3.lBS | assoc | M |
| *Tyrannosaurus^3^* | Fisk | HC-235 | MOR-2701 | sls | L3.uBS | assoc | S |
| *Edmontosaurus* | Joe’s Duck | HC-XO4 | MOR-P04.1 | ss | L3.mMS | **artic** | M |
| *Edmontosaurus^3^* | Angie's Foot | HC-629 | MOR-3001 | sls | L3.lMS | assoc | L |
| *Edmontosaurus* | Jack's Leg | HC-241 | none | sls | L3.uBS | assoc | L |
| *Edmontosaurus* | Ugly Duck | HC-245 | MOR-1123 | ss | L3.mBS | assoc | L |
| *Edmontosaurus^3^* | Becky’s Giant | HC-380 | MOR-1609 | ss | L3.mBS | assoc | XL |
| *Edmontosaurus* | X-rex | HC-248 | MOR-1142 | ss | L3.lBS | **artic** | XL |
| *Thescelosaurus* | Thescelo tail | HC-X00.1 | MOR.P00.1 | ms | L3.uMS | **artic** | L |
| *Thescelosaurus* | Arrowhead | HC-248 | MOR-1106 | ms | L3.uMS | assoc | L |
| *Thescelosaurus* | Heaven | HC-285 | MOR-1165 | ms | L3.lMS | assoc | M |
| *Thescelosaurus* | Harmon#1 | HC-X01.1 | MOR.P01.1 | ms | L3.uBS | assoc | M |
| *Ornithomimus* | KOS | HC-268 | MOR-1134 | sls | L3.uMS | assoc | M |
| *Ornithomimus* | Surf & Turf | HC-246 | MOR-1104 | sls | L3.uBS | assoc | M |
| *Ornithomimus* | F-9 | HC-306 | MOR-1181 | sls | L3.uBS | **artic** | M |
| *Ornithomimus* | Many Toes | HC-311 | MOR-1189 | ss | L3.uBS | assoc | L |
| *Ornithomimus* | FOS | HC-247 | MOR-1105 | sls | L3.lBS | assoc | L |
| *Ankylosaurus* | Angi's Ank | HC-284 | MOR-1159 | sls | L3.uBS | assoc | L? |
| *Ankylosaurus^3^* | Mark's Ank | HC-315 | MOR-1197 | ss | L3.lBS | assoc | L? |
| Tables S1 – S6 list meta-data recorded in this dinosaur census by taxon and stratigraphic level with MOR locality name, MOR locality number (HC-xxx), MOR specimen catalog number (MOR-xxx), sedimentology, stratigraphic subdivision and facies, associated or articulated skeletal preservation, and relative ontogenetic stage (S-XL; A-F as defined under Census Methods). Abbreviations: assoc, associated; artic, articulated; HC-xxx, Hell Creek locality number; loc., locality; MOR, Museum of the Rockies; MOR-xxx, MOR specimen number format; ms, mudstone; no., number; Onto., ontogenetic stage; Pres., skeletal preservation; Sed., sedimentology; sls, siltstone; ss, sandstone; Strat., stratigraphic unit and facies.  Ontogenetic abbreviations: S, small; M, medium; L, large; XL, extra-large; A-E, six ontogenetic stages identified by ascending size from “A” to “F”. *Triceratops* has a relatively wide ontogenetic with higher ontogenetic resolution than other dinosaur taxa. Each letter corresponds to a relative age class: “A,” represents small juveniles; “B,” large juveniles; “C,” small subadults; “D,” large subadults; “E,” small adults; and “F,” large adults. These stages are simply determined relative to one another based on the smallest and largest end member skulls. Within specimens of *Triceratops*, for example, “A” individuals have skulls approximately 0.3 m in length; F-size skulls are approximately 3.0 m in length. Letters in-between “A” and “F” provide a general sense of intervening sizes. Four size ranges are utilized for taxa other than *Triceratops* and for isolated fossils from lag deposits. S, small; M, medium; L, large; XL, extra large. These size classes are completely subjective, but useful for the purpose of demonstrating the rarity or abundance of certain sizes of individuals and communicating these occurrences.  Stratigraphic abbreviations in the lower Hell Creek Formation (L3): L3lBS, lower basal sandstone; L3.mBS, middle basal sandstone; L3.uBS, upper basal sandstone; L3.lMS, lower mudstone; L3.mMS, middle mudstone; L3.uMS, upper mudstone; and L3BL, 3B1 lag at base of Jen-rex sand.  Seven specimens with superscript^3^ consisted of only three elements each. Limited excavation around the elements failed to yield more material and the sites were abandoned after the collection was made. | | | | | | | |
