# Supplementary material for: Dinosaur Census Reveals Abundant Tyrannosaurus and Rare Ontogenetic Stages in the Upper Cretaceous Hell Creek Formation (Maastrichtian), Montana, USA
Source: PLoS One. 2011 Feb 9;6(2):e16574. doi: 10.1371/journal.pone.0016574 (PMC3036655; doi:10.1371/journal.pone.0016574)
Supplement: Table S2 — Upper Hell Creek Formation (U3) Triceratops skulls collected. These specimens are either whole skulls or isolated elements and do not qualify to be counted under the “three bone rule.” (DOC) [file pone.0016574.s002.doc]

| **Record No.** | **Locality Name** | **Date Found** | **Sediment** | **Stratigraphic unit & facies** |
| --- | --- | --- | --- | --- |
| Trike 7 | Bob's Trike | 7.6.99 | mudstone | L3.lMS |
| Trike 21 | CCB | 7.19.99 | mudstone | L3.lMS |
| Trike 22 | Shell-Bed Trike | 7.19.99 | siltstone | L3.uBS |
| Trike 36 | Mark's Trike | 7.31.99 | mudstone | L3.lMS |
| Trike 37 | Bob's Trike | 7.31.99 | mudstone | L3.lMS |
| Trike 38 | Weird Micro | 8.14.99 | siltstone | L3.uBS |
| Trike 43 | H & F Trike | 8.19.00 | mudstone | L3.lMS |
| Trike 51 | BH-6.12.00.7 | 6.12.00 | mudstone | L3.lMS |
| Trike 52 | Jack's 6.12 Trike1 | 6.12.00 | mudstone | L3.lMS |
| Trike 53 | Jack's 6.12 Trike2 | 6.12.00 | sandstone | L3.mBS |
| Trike 60 | Larry's Toe | 7.2.00 | mudstone | L3.lMS |
| Trike 63 | Larry's 7.2 Trike | 7.2.00 | mudstone | L3.lMS |
| Trike 78 | Jack's 7.8 Trike | 7.8.00 | mudstone | L3.lMS |
| Trike 86 | Mark's 7.17 Trike | 7.17.00 | mudstone | L3.lMS |
| Trike 91 | ATV.7.20 | 7.20.00 | mudstone | L3.mMS |
| Trike 92 | Cold Turkey Trike | 7.20.00 | mudstone | L3.lMS |
| Trike 104 | Doug's Saddle | 7.28.00 | mudstone | L3.lMS |
| Trike 120 | Danny's Trike | 8.1.00 | mudstone | L3.mMS |
| Trike 126 | Celeste's 8.16 Trike | 8.16.00 | mudstone | L3.lMS |
| Trike 127 | Bob & I | 8.22.00 | siltstone | L3.mMS |
| Trike 128 | Larry's 8.23 Trike | 8.23.00 | siltstone | L3.mMS |
| Trike 146 | Dead Tree | 7.5.01 | mudstone | L3.lMS |
| Trike 151 | Clams & Trike | 7.20.01 | siltstone | L3.mBS |
| Trike 156 | DK Site | 7.25.01 | mudstone | L3.lMS |
| Trike 160 | Goodwin 8.2 | 8.2.01 | siltstone | L3.uBS |
| Trike 163 | Horner 8.5-2 | 8.5.01 | mudstone | L3.lMS |
| Trike 164 | Horner 8.5-1 | 8.5.01 | siltstone | L3.lMS |
| Trike 167 | Trike BC | 8.5.01 | mudstone | L3.lMS |
| Trike 168 | Blown-Up | 8.6.01 | mudstone | L3.lMS |
| Trike 170 | Imploded Trike | 8.6.01 | mudstone | L3.lMS |
| Trike 171 | Laura Hideaway | 8.6.01 | mudstone | L3.lMS |
| Trike 174 | Bandana | 8.6.01 | mudstone | L3.lMS |
| Trike 175 | JRH-17 | 8.6.01 | mudstone | L3.lMS |
| Trike 178 | Heaven | 8.10.01 | mudstone | L3.mMS |
| Trike 192 | Scott's BD | 5.26.02 | mudstone | L3.lMS |
| Trike 203 | HK-4 | 7.15.02 | mudstone | L3.lMS |
| Trike 214 | V. Bros. Trike | 8.2.02 | sandstone | L3.uBS |
| Trike 215 | Mark's Baby Trike | 8.3.02 | mudstone | L3.lMS |
| Trike 218 | Trash Trike | 8.3.02 | mudstone | L3.mMS |
| Trike 225 | Apex Trike | 6.15.03 | siltstone | L3.mMS |
| Trike 241 | CC's Trike | 6.18.04 | mudstone | L3.lMS |
| Trike 247 | Becky's Trike | 6.20.04 | sandstone | L3.mBS |
| Trike 250 | Cut Throat | 6.23.04 | mudstone | L3.mMS |
| Trike 255 | Blown Trike | 7.24.04 | mudstone | L3.lMS |
| Trike 257 | CJ Trike | 7.25.04 | mudstone | L3.lMS |
| Trike 259 | C & J Trike | 7.28.04 | mudstone | L3.uMS |
| These *Triceratops* skulls remain in the field and were not included in this census because it could not be confirmed if three or more disarticulated bones were present without extensive excavation due to poor preservation. While a “fused” skull may be composed of more than three bones, it cannot be determined with certainty that it is not reworked and these specimens were not counted in the census. In later years of the Hell Creek Project, time was invested and excavations undertaken to confirm the status of all *Triceratops* specimens. Abbreviations: see Table S1. | | | | |
