# Supplementary material for: Dinosaur Census Reveals Abundant Tyrannosaurus and Rare Ontogenetic Stages in the Upper Cretaceous Hell Creek Formation (Maastrichtian), Montana, USA
Source: PLoS One. 2011 Feb 9;6(2):e16574. doi: 10.1371/journal.pone.0016574 (PMC3036655; doi:10.1371/journal.pone.0016574)
Supplement: Table S3 — Dinosaur census from 3B1 lag deposit (MOR loc. HC-312) at the base of the Jen-rex sand in order of abundance. (DOC) [file pone.0016574.s003.doc]

| **Field No.** | **Taxon** | **Element** | **Size** |
| --- | --- | --- | --- |
| HC-312.B3 | *Triceratops* | caudal | S |
| HC-312.I | *Triceratops* | dorsal vertebra | M |
| HC-312.Y | *Triceratops* | quadratojugal | M |
| HC-312.QQ | *Triceratops* | basioccipital | M |
| HC-312.RR | *Triceratops* | postorbital horn | M |
| HC-312.B | *Triceratops* | braincase fragment | L |
| HC-312.C | *Triceratops* | caudal | L |
| HC-312.F | *Triceratops* | occipital condyle | L |
| HC-312.H | *Triceratops* | manus phalange | L |
| HC-312.U | *Triceratops* | distal fibula | L |
| HC-312.X | *Triceratops* | pes ungual | L |
| HC-312.Z | *Triceratops* | caudal vertebra | L |
| HC-312.EE | *Triceratops* | jugal boss | L |
| HC-312.GG | *Triceratops* | frill fragment | L |
| HC-312.HH | *Triceratops* | postorbital horn fragment | L |
| HC-312.MM | *Triceratops* | distal metatarsal | L |
| HC-312.OO | *Triceratops* | quadratojugal | L |
| HC-312.ZZ | *Triceratops* | jaw fragment | L |
| HC-312.A4 | *Triceratops* | basioccipital | L |
| HC-312.A6 | *Triceratops* | pes phalange | L |
| HC-312.B1 | *Triceratops* | frill fragment | L |
| HC-312.Q | *Triceratops* | basisphenoid | XL |
| HC-312.T | *Triceratops* | occipital condyle | XL |
| HC-312.N | *Tyrannosaurus* | metatarsal | S |
| HC-312.JJ | *Tyrannosaurus* | distal pubis | S |
| HC-312.A7 | *Tyrannosaurus* | distal metatarsal | S |
| HC-312.J | *Tyrannosaurus* | pes phalange | M |
| HC-312.W | *Tyrannosaurus* | pes phalange | M |
| HC-312.DD | *Tyrannosaurus* | pes phalange | M |
| HC-312.A | *Tyrannosaurus* | caudal | L |
| HC-312.E | *Tyrannosaurus* | maxilla fragment | L |
| HC-312.L | *Tyrannosaurus* | pes phalange | L |
| HC-312.R | *Tyrannosaurus* | fibula | L |
| HC-312.AA | *Tyrannosaurus* | distal metatarsal | L |
| HC-312.FF | *Tyrannosaurus* | manus ungual | L |
| HC-312.LL | *Tyrannosaurus* | tooth | L |
| HC-312.XX | *Tyrannosaurus* | tooth | L |
| HC-312.A3 | *Tyrannosaurus* | tibia shaft fragment | L |
| HC-312.B4 | *Tyrannosaurus* | supraorbital | L |
| HC-312.B5 | *Tyrannosaurus* | surangular | L |
| HC-312.B7 | *Tyrannosaurus* | distal caudal | L |
| HC-312.B9 | *Tyrannosaurus* | caudal centrum | L |
| HC-312.BB | *Edmontosaurus* | maxilla | M |
| HC-312.II | *Edmontosaurus* | caudal vertebra | M |
| HC-312.TT | *Edmontosaurus* | distal ulna | M |
| HC-312.UU | *Edmontosaurus* | pes ungual | M |
| HC-312.WW | *Edmontosaurus* | squamosal | M |
| HC-312.B2 | *Edmontosaurus* | maxilla fragment | M |
| HC-312.G | *Edmontosaurus* | maxilla | L |
| HC-312.K | *Edmontosaurus* | caudal vertebra | L |
| HC-312.V | *Edmontosaurus* | pes phalange | L |
| HC-312.S | *Edmontosaurus* | maxilla | L |
| HC-312.PP | *Edmontosaurus* | manus ungual | L |
| HC-312.O | *Edmontosaurus* | pes ungual | L |
| HC -312.P | *Edmontosaurus* | pes phalange | L |
| HC-312.A2 | *Edmontosaurus* | manus phalange | L |
| HC-312.A5 | *Edmontosaurus* | jaw fragment | L |
| HC-312.A8 | *Edmontosaurus* | pes phalange | L |
| HC-312.A9 | *Edmontosaurus* | distal radius | L |
| HC-312.NN | *Edmontosaurus* | pes phalange | XL |
| HC-312.SS | *Thescelosaurus* | pes phalange | M |
| HC-312.VV | *Thescelosaurus* | pes phalange | M |
| HC-312.B8 | *Thescelosaurus* | dorsal vertebra | M |
| HC-312.D | *Thescelosaurus* | distal femur | L |
| HC-312.M | *Thescelosaurus* | pes phalange | L |
| HC-312.KK | *Thescelosaurus* | caudal centrum | L |
| HC-312.YY | *Thescelosaurus* | pes phalange | L |
| HC-312.A1 | *Ornithomimus* | pes phalange | L |
| HC-312.B6 | *Ornithomimus* | pes phalange | L |
| HC-312.CC | *Pachycephalosaurus* | dorsal vertebra | L |
| Abbreviations: size designations S, M, L, XL defined in Table S1. | | | |
