# Supplementary material for: Dinosaur Census Reveals Abundant Tyrannosaurus and Rare Ontogenetic Stages in the Upper Cretaceous Hell Creek Formation (Maastrichtian), Montana, USA
Source: PLoS One. 2011 Feb 9;6(2):e16574. doi: 10.1371/journal.pone.0016574 (PMC3036655; doi:10.1371/journal.pone.0016574)
Supplement: Table S4 — Upper Hell Creek Formation (U3) skeletons in order of abundance. (DOC) [file pone.0016574.s004.doc]

| **Taxon** | **Locality Name** | **Loc. No.** | **MOR No.** | **Sed.** | **Strat.** | **Pres.** | **Onto.** |
| --- | --- | --- | --- | --- | --- | --- | --- |
| *Triceratops* | Juvie Trike III | HC-544 | MOR-2951 | ms | U3.ibMS | assoc | B |
| *Triceratops* | SG-5 | HC-250 | MOR-1110 | ms | U3.ibMS | assoc | C |
| *Triceratops* | Quittin Time | HC-443 | MOR-2574 | ms | U3.ibMS | assoc | D |
| *Triceratops* | Lazy Bones | HC-528 | MOR-2936 | ms | U3.ibMS | assoc | D |
| *Triceratops* | Prospero | HC-529 | MOR-2937 | ms | U3.ibMS | assoc | D |
| *Triceratops* | Crazy Trike | HC-564 | MOR-2971 | ms | U3.ibMS | assoc | D |
| *Triceratops* | Situ But Sad | HC-627 | MOR-2999 | ms | U3.ibMS | assoc | D |
| *Triceratops** | MORT | HC-135 | MOR-004 | ms | U3.ibMS | **artic** | E |
| *Triceratops* | Engdahl Trike | HC-196 | MOR-622 | ms | U3.ibMS | **artic** | E |
| *Triceratops* | Haxby | HC-385 | MOR-1625 | ss | U3.AS | assoc | E |
| *Triceratops* | Legs | HC-566 | MOR-2974 | ss | U3.AS | assoc | E |
| *Triceratops* | City Slicker 2 | HC-392 | MOR-2551 | ms | U3.ibMS | assoc | E |
| *Triceratops* | Mark's Trike 2 | HC-444 | MOR-2597 | ms | U3.ibMS | assoc | E |
| *Triceratops* | Lon's Trike | HC-521 | MOR-2924 | ss | U3.AS | assoc | E |
| *Triceratops* | Lauren's Trike | HC-531 | MOR-2938 | ms | U3.ibMS | assoc | E |
| *Triceratops* | Homer's Nose | HC-545 | MOR-2952 | ms | U3.ibMS | assoc | E |
| *Triceratops* | Ashes Trike | HC-628 | MOR-3000 | ms | U3.ibMS | assoc | E |
| *Triceratops* | Devil's Horns | HC-643 | MOR-3016 | ms | U3.ibMS | assoc | E |
| *Triceratops* | Quittin Time | HC-443 | MOR-2702 | ms | U3.ibMS | assoc | F |
| *Triceratops* | Super Nasal | HC-565 | MOR-2972 | ss | U3.AS | assoc | F |
| *Triceratops* | More Frill | HC-567 | MOR-2975 | ms | U3.ibMS | assoc | ? |
| *Triceratops* | Seth's Trike | HC-571 | MOR-2979 | ms | U3.ibMS | assoc | ? |
| *Triceratops* | PVU Trike | HC-x09.1 | MOR-P09.1 | sls | U3.ibMS | assoc | ? |
| *Tyrannosaurus* | 4H24 | HC-492 | MOR-2708 | ms | U3.ibMS | assoc | S |
| *Tyrannosaurus* | Hager | HC-139 | MOR-009 | ss | U3.10mS | assoc | M |
| *Tyrannosaurus* | H-rex | HC-319 | MOR-1602 | ss | U3.ibMS | assoc | M |
| *Tyrannosaurus* | Wankel | HC-069 | MOR-555 | ss | U3.10mS | **artic** | L |
| *Tyrannosaurus* | Blown T | recorded | uncollected | ss | U3.AS | assoc | L |
| *Edmontosaurus* | Sleepy Hollow | HC-x09.2 | MOR-P09.2 | ss | U3.AS | **artic** | M |
| *Edmontosaurus* | Tom's Old Duck | HC-631 | MOR-3002 | ss | U3.10mS | assoc | M |
| *Edmontosaurus^3^* | Duckleberry | HC-245 | MOR-1103 | sls | U3.ibMS | assoc | L |
| *Edmontosaurus* | Ducky Tail | HC-532 | MOR-2939 | ss | U3.10mS | **artic** | L |
| *Edmontosaurus* | Edmonto Tail | HC-137 | MOR-007 | ss | U3.ibMS | **artic** | XL |
| Abbreviations: artic, articulated skeleton; assoc, associated skeleton; AS, Apex sandstone; ms, mudstone; sl, siltstone; ss, sandstone; U3, upper Hell Creek Formation; ibMS, interbedded variegated mudstone; 10mS, 10 meter sandstone; Loc. no., locality number; MOR no., MOR specimen number; onto, ontogenetic stages as defined in Table S1; Pres., preservation; Sed., sedimentology; Strat., stratigraphic unit and facies. *Triceratops** (MORT) composed of two elements: a taphonomically “fused” skull and lower jaws. *Edmontosaurus^3^* found with only three elements and limited excavation did not yield additional fossils. | | | | | | | |
