# Supplementary material for: Dinosaur Census Reveals Abundant Tyrannosaurus and Rare Ontogenetic Stages in the Upper Cretaceous Hell Creek Formation (Maastrichtian), Montana, USA
Source: PLoS One. 2011 Feb 9;6(2):e16574. doi: 10.1371/journal.pone.0016574 (PMC3036655; doi:10.1371/journal.pone.0016574)
Supplement: Table S5 — Upper Hell Creek Formation (U3) Triceratops skulls recorded but not collected. (DOC) [file pone.0016574.s005.doc]

| **Record No.** | **Site Name** | **Date Collected** | **Sedimentology** | **Stratigraphic Unit** |
| --- | --- | --- | --- | --- |
| Trike U3 | WP-005 | 7.2.05 | sandstone | U3.10mS |
| Trike U4 | Meaningless | 6.7.06 | mudstone | U3.ibMS |
| Trike U5 | Funky Chunks | 7.7.06 | mudstone | U3.ibMS |
| Trike U6 | Chance's Trike | 7.13.07 | mudstone | U3.ibMS |
| Trike U7 | Malorie's Trike | 7.13.07 | mudstone | U3.ibMS |
| Trike U8 | Nosey Nasal | 7.17.07 | sandstone | U3.10mS |
| Trike U9 | Joe's Trike | 7.23.07 | sandstone | U3.10mS |
| Trike U10 | Trike Basin | 8.1.07 | siltstone | U3.ibMS |
| Trike U11 | JRH-061 | 7.19.08 | mudstone | U3.ibMS |
| Trike U12 | JRH-062 | 7.20.08 | mudstone | U3.ibMS |
| Trike U13 | Billy Jean | 6.12.09 | mudstone | U3.ibMS |
| Trike U14 | GPS Maniac | 7.7.09 | siltstone | U3.ibMS |
| Trike U15 | Orange Peel | 7.12.09 | mudstone | U3.ibMS |
| Trike U16 | Trike Pants | 7.15.09 | mudstone | U3.ibMS |
| Trike U17 | Ol Rusty | 7.29.09 | siltstone | U3.ibMS |
| Trike U18 | Devil's Horns | 8.1.09 | mudstone | U3.ibMS |
| Abbreviations: U3, upper Hell Creek Formation; 10mS, 10 meter sandstone; ibMS, interbedded variegated mudstone. | | | | |
