# Supplementary material for: Dinosaur Census Reveals Abundant Tyrannosaurus and Rare Ontogenetic Stages in the Upper Cretaceous Hell Creek Formation (Maastrichtian), Montana, USA
Source: PLoS One. 2011 Feb 9;6(2):e16574. doi: 10.1371/journal.pone.0016574 (PMC3036655; doi:10.1371/journal.pone.0016574)
Supplement: Table S6 — Census from the Doldrum's lag deposit (MOR loc. HC-530) at the base of the Apex sandstone in the upper Hell Creek Formation (U3.AS) in order of abundance. (DOC) [file pone.0016574.s006.doc]

| **Field No.** | **Taxon** | **Element** | **Size** |
| --- | --- | --- | --- |
| HC-530.H | *Triceratops* | basioccipital | M |
| HC-530.L | *Triceratops* | pes ungual | M |
| HC-530.KK | *Triceratops* | nasal | M |
| HC-530.A | *Triceratops* | occipital condyle | L |
| HC-530.C | *Triceratops* | caudal | L |
| HC-530.D | *Triceratops* | caudal | L |
| HC-530.J | *Triceratops* | caudal | L |
| HC-530.K | *Triceratops* | metacarpal | L |
| HC-530.P | *Triceratops* | caudal | L |
| HC-530.Q | *Triceratops* | coracoid | L |
| HC-530.AA | *Triceratops* | postorbital horn | L |
| HC-530.MM | *Triceratops* | neural spine | L |
| HC-530.OO | *Triceratops* | squamosal | L |
| HC-530.PP | *Triceratops* | postorbital sinus | L |
| HC-530.RR | *Triceratops* | basioccipital | L |
| HC-530.SS | *Triceratops* | squamosal fragment | L |
| HC-530.O | *Tyrannosaurus* | dorsal vertebra | S |
| HC-530.HH | *Tyrannosaurus* | astragulus | S |
| HC-530.DD | *Tyrannosaurus* | pes ungual | M |
| HC-530.II | *Tyrannosaurus* | exoccipital | M |
| HC-530.BB | *Tyrannosaurus* | fibula fragment | L |
| HC-530.QQ | *Tyrannosaurus* | maxilla fragment | L |
| HC-530.TT | *Tyrannosaurus* | jaw fragment | L |
| HC-530.UU | *Tyrannosaurus* | caudal | L |
| HC-530.VV | *Tyrannosaurus* | jaw fragment | L |
| HC-530.B | *Edmontosaurus* | pes phalange | M |
| HC-530.I | *Edmontosaurus* | sacral segment | M |
| HC-530.V | *Edmontosaurus* | pes phalange | M |
| HC-530.W | *Edmontosaurus* | dentary | M |
| HC-530.Y | *Edmontosaurus* | pes phalange | M |
| HC-530.NN | *Edmontosaurus* | maxilla | M |
| HC-530.LL | *Edmontosaurus* | quadrate | L |
| HC-530.E | *Thescelosaurus* | pes ungual | M |
| HC-530.M | *Thescelosaurus* | dorsal vertebra | L |
| HC-530.N | *Thescelosaurus* | caudal | L |
| HC-530.S | *Thescelosaurus* | pes phalange | L |
| HC-530.EE | *Ornithomimus* | metatarsal | M |
| HC-530.CC | *Ornithomimus* | pes phalange | L |
| HC-530.JJ | *Pachycephalosaurus* | cranial | S |
| Size abbreviations: same as defined in Table S1. | | | |
